# The Role of Tumor Microenvironment and Immune Response in Colorectal Cancer Development and Prognosis

**DOI:** 10.3389/pore.2022.1610502

**Published:** 2022-07-21

**Authors:** Maria Wozniakova, Jozef Skarda, Milan Raska

**Affiliations:** ^1^ Institute of Pathology and Molecular Genetics, University Hospital Ostrava, Ostrava, Czechia; ^2^ Department of Clinical and Molecular Pathology, Faculty of Medicine and Dentistry, Palacký University Olomouc, Olomouc, Czechia; ^3^ Department of Immunology, Faculty of Medicine and Dentistry, Palacký University Olomouc, Olomouc, Czechia

**Keywords:** colorectal cancer, tumor microenvironment, immune cells, tumorigenesis, consensus molecular subtypes, immunoscore

## Abstract

Colorectal cancer (CRC) is one of the most common cancers worldwide. The patient’s prognosis largely depends on the tumor stage at diagnosis. The pathological TNM Classification of Malignant Tumors (pTNM) staging of surgically resected cancers represents the main prognostic factor and guidance for decision-making in CRC patients. However, this approach alone is insufficient as a prognostic predictor because clinical outcomes in patients at the same histological tumor stage can still differ. Recently, significant progress in the treatment of CRC has been made due to improvements in both chemotherapy and surgical management. Immunotherapy-based approaches are one of the most rapidly developing areas of tumor therapy. This review summarizes the current knowledge about the tumor microenvironment (TME), immune response and its interactions with CRC development, immunotherapy and prognosis.

## Introduction

Uncontrollable proliferation and metastasis of cancer cells within major organs, such as the liver and lung, constitute the leading causes of death in CRC patients (1). Treatment options for CRC are complex, but it must be emphasized that only some patients can undergo radical treatment of metastases in the sense of resection with curative intent. The treatment is usually based on combined chemotherapy targeting primarily the vascular endothelial growth factor receptor (VEGFR) or the epidermal growth factor (epidermal growth factor receptor–EGFR) signaling pathways (2). Unfortunately, not all patients can be treated by these selective drugs, especially EGFR inhibitors. Modern immunotherapy methods significantly expand the possibilities of treatment for patients suffering from this serious disease. This review summarizes the current knowledge about the tumor TME and the immune processes involved in the development, growth and prognosis of CRC.

## Methods

We searched for keywords “immunotherapy colorectal cancer”, “tumor infiltrating lymphocytes colorectal cancer” and “PD-L1 colorectal cancer” in the database PubMed, which gave us a total of 794 studies. After discarding duplicates, we obtained 320 studies that were scanned. We excluded 240 studies unrelated to the topic, thus acquiring 65 studies that were used as a basis for this review. Further 44 papers were identified by cross-referencing.

### Colorectal Cancer Tumorigenesis

Three major pathways of CRC tumorigenesis have been thoroughly described: chromosomal instability (CIN), microsatellite instability (MSI), and the CpG island methylator phenotype (CIMP) pathway (3). The tumor mutation burden (TMB) is an emerging biomarker in multiple tumor types. Highly mutated tumors are thought to harbor an increased neoantigen burden, making them immunogenic and responsive to immunotherapy (4). CIN is the most common type of genetic instability, present in 70%–85% of CRC, impacting chromosome copy number and structure (5). The CIMP pathway may contribute to silencing the tumor suppressor and DNA repair genes due to hypermethylation (6). The prevalence of CIMP varied from 21% to 27% cases of CRC across geographical regions. In some patients, both pathways may coexist; the most common combination is that of CIMP and MSI (7).

The third pathway, MSI, is a result of DNA mismatch repair deficiency (dMMR) caused by the inactivation of genes encoding proteins responsible for repairing DNA errors, mainly occurring during replication. The prognosis of tumors developing through MSI pathway is better than that of other pathways (8). Please note that the terms MSI tumors and dMMR tumors are usually used interchangeably and they will be used in this way also in this paper, with the reference to MMR preferred when speaking of the pathway and the term MSI preferred when speaking of the type of tumor. According to established guidelines, MSI is determined by a panel of microsatellite markers, commonly mononucleotides (9). Microsatellites are short repetitive DNA sequences of uniform length containing one to six bases. They are located in the non-coding regions of the genome and, less frequently, they were also identified in the regulatory or coding regions. They are highly polymorphic between subjects, but within an individual, their length is stable and uniform (10). dMMR CRCs produce a higher number of neoantigen types than MMR-proficient ones, which, consequently, leads to the greater activation of the immune system (11). MMR deficiency has been detected in approximately 15%–20% of CRC (12, 13). The sporadic microsatellite-unstable phenotype usually results from epigenetic silencing of the mismatch repair gene mutL homolog 1 (MLH1). Germline mutations of one of the broader groups of mismatch repair genes (MLH1, MSH2, MSH6, and PMS2) are typical of hereditary Lynch syndrome (14). MSI tumors are often poorly differentiated with mucinous features or a medullary growth pattern, although they can also resemble more typical CRCs with tubular or cribriform growth patterns and less pronounced mucus formation (15). Patients with MSI tumors have a more favorable prognosis than those with MS-stable tumors, which has been explained by the generally strong cytotoxic CD45RO + lymphocyte infiltration resulting from high TMB and consequently high neoantigen load (16–18). Densities of CD3^+^ and CD8^+^ cells are higher in this group of tumors, but the densities of CD4^+^ and transcription factor forkhead box P3 (Foxp3)+ cells were not affected by the MSI status of the tumor (15, 19).

## The Role of the Immune System in the Prognosis and Treatment of Colorectal Cancer

The immune system plays an important role in cancer control and treatment response and the presence and quantity of key immune cell subtypes within the TME of CRC are known to possess prognostic potential (20). The phenotype and localization of various immune cell types (B and T cells, natural killer cells, tumor-associated macrophages and dendritic cells, myeloid-derived suppressor cells, mast cells and cancer-associated fibroblasts) in individual areas of the TME also constitute important histopathological features of prognostic value (11). Besides, immune checkpoint inhibitors, including cytotoxic T-lymphocyte-associated antigen 4 (CTLA-4) inhibitors, programmed cell death 1 (PD-1) and programmed cell death ligand 1 (PD-L1) inhibitors have been associated with significant improvement in various advanced cancers (21, 22). The role of these factors will be discussed individually below.

### Role of CTLA-4 in Colorectal Cancer

CTLA-4 is considered the “leader” of immune checkpoints as it stops potentially autoreactive T cells as soon as the initial stage of naïve T-cell activation, typically in lymph nodes (21). CTLA-4 blockade was shown to inhibit tumor progression by increasing the effector T-cells activity and suppressing regulatory T cells (Treg cells) (23). The Tregs population was associated with cancer progression and metastasis. In hepatocellular carcinoma patients, for example, Han et al. identified tumor-infiltrating human CD4^+^CD69^+^ Tregs capable of suppressing CD4^+^ T cell response via membrane-bound transforming growth factor (TGF)-β1; the percentage of these cells significantly correlated with tumor progression (24). It is important to realize that Tregs have also the ability to inhibit the T cell-mediated immune response against tumor cells in CRC (25–27). Xu et al. reported that the function of tumor-infiltrating Tregs in a cancer nest differs from their functions in cancer stroma. In the latter, they act predominantly as inhibitors of inflammatory anti-microbial response; in a cancer nest, however, they act as suppressors of the anti-tumor response (28). The improved understanding of the detailed mechanism could help us develop new immunotherapy methods targeting CTLA-4 Tregs, ideally in specific locations.

### PD-1 and PD-L1 Pathway in Colorectal Cancer

A core concept in cancer immunotherapy is that tumor cells, which would normally be recognized by T cells, have developed ways to evade the host immune system by taking advantage of peripheral tolerance and hiding from immune recognition (29). The PD-1 pathway regulates previously activated T cells, primarily in peripheral tissues (30). Takasu et al. positively correlated the activity of the PD-1 pathway with the expression of Foxp3, which is widely known to be involved in the development and function of Treg cells including CD4^+^CD25^+^ and CD8^+^CD25^+^ cells (31, 32). Several studies have confirmed that high Foxp3 expression in Treg cells allows tumor cells in various types of human carcinomas to escape from immunological surveillance and, in this way, promotes their proliferation and tumor development (33, 34). Foxp3 can be expressed also by cancer cells, which results in the secretion of immunosuppressive cytokines such as IL-10 and TGFβ into the tumor microenvironment, which might allow them to evade effector T-cell responses and, hence, to progress (35). On the other hand, Yoon et al. and Ling et al. suggest that high Foxp3 T-cell density is a favorable prognostic factor in CRCs with low cytotoxic CD8^+^ infiltration (36, 37). This paradox is probably caused by dense microbiological flora present in the large intestine. The colon is constantly confronted with foreign antigens, which leads to increased levels of Foxp3 T-cells even in a physiological state. A high amount of Treg cells, suppressing microbially induced inflammation, could act protectively not only by this prevention of the inflammatory response that can potentially lead to the development of cancer in the colorectal epithelium but also by preventing tumor growth; this could make it an exception to the rule of the poor prognostic role of high Foxp3+ expression (37, 38).

PD-1 and PD-L1 are crucial checkpoints of the immune system response. They provide negative feedback that inhibits T helper 1 (Th1) cytotoxic immune responses, causing T-cell exhaustion or tolerance (39). PD-L1 is constitutively expressed by T and B cells, dendritic cells, and macrophages (40), as well as by other cell types including endothelial, pancreatic, and muscle cells (41). Expression of PD-L1 either in the tumor or in the infiltrating immune cells has been verified (predominantly by immunohistochemistry) in a variety of tumors, suggesting its role as a prognostic trait and therapeutic target across multiple types of tumors. The overexpressed PD-L1 on the surface of cancer cells binds to the PD-1 on tumor-infiltrating lymphocytes (TILs), which counteracts the TCR-signaling cascade by phosphorylating SHP-2 and as a result, impairs T cell activation (42). This process is an analogy to the interaction of CD28 with the B7 molecules (CD80 or CD86) on professional antigen-presenting cells (APC); the interruption of this signaling pathway with CD28 antagonists can be typically considered a positive effect inducing suppression of the pathological immune response; in some cases, however, it can also negatively affect the organism by inducing the antigen-specific tolerance (43).

Recent reports indicated that while PD-L1 expression on CRC tumor cells was commonly associated with poor outcome or was not prognostic, lymphocytic expression of PD-L1 and PD-1 in CRC was associated with good clinical outcome, particularly in certain colon locations (44, 45). PD-1 and PD-L1 expressions are regulated by numerous factors, including inflammatory stimuli (IFN, IL-6, TNF) and oncogenic pathways at transcription, post-transcription, and post-translation (e.g., EGFR, MAPK, JAK-STAT pathway) levels (46).

Inhibition of the PD-1/PD-L1 signaling is a feasible strategy for normalizing the TME and up to now, this approach has been used in the treatment of various cancers, such as melanoma, non-small cell lung cancer, gastric cancer, renal cancer, liver cancer, urothelial cancer, lymphoma, and all microsatellite instability-high (MSI-H) cancers (47). Noh et al. hypothesized that PD-L1 overexpression in CRC tumor cells is associated with broader activation of CD8^+^ T cells, which leads, among other things, to enhanced local expression of supporting factors such as IFN-γ. Enhanced CD8 recognition of CRC cells could be also linked to increased or aberrant expression of normal proteins due to the increased number of nonsynonymous, somatic mutations and MSI in tumor cells (48, 49). Practically, the expression of PD-L1 is primarily attributed to tumor-associated immune cells, most prominent at the tumor-stroma interface along the invasive margin. The true expression of PD-L1 by a tumor cell is a rare event (50).

Clinicopathologically, PD-L1 positivity, as well as PD-1 density, tend to associate with proximal tumor location and poor differentiation (51), and these features are commonly seen in MSI-H CRCs. On the contrary, mismatch repair-proficient tumors have usually high budding and show a low PD-L1 and PD-1 positivity (52). Generally, the low number of PD-L1 positive tumor samples may suggest that the PD-1/PD-L1 checkpoint mechanism is not the major escape mechanism for tumor cells in CRC, which could also explain the limited impact of anti-PD-L1/PD-1 therapy in this type of cancer (53). Shen et al. suggested that the expression of PD-L1 could be utilized as an independent factor in judging the prognosis of CRC, and reported that patients with advanced cancer or lymphatic invasion were more likely to express PD-L1 (54).

According to recent resources, the expression of PD-L1 in immune cells is significantly higher in MSI CRCs when compared to non-MSI tumors (50), which is believed to be one of the reasons for the better prognosis of these tumors (55). Ganesh et al. suggest that PD-1 blocking therapy potentially benefits patients with MMR/MSI subsets of CRCs (56). The reported overall objective response rate was 36% (51). In 2017, the PD-1 inhibitor Pembrolizumab was approved by the US Food and Drug Administration for treatment of metastatic solid MSI tumors, including CRC (57). Nevertheless, it has been speculated that with disease progression and development of metastases, dMMR/MSI tumors evade host immune surveillance, which is in turn associated with a loss of the prognostic advantage. This is seen in stage IV CRCs with dMMR/MSI where no prognostic advantage was found (58). Unfortunately, as MSI tumors represent only about 15 % of CRCs, the monotherapy with immune checkpoint inhibitors has shown only limited efficacy when applied to the general CRC population (53). All these facts indicate that the function of PD-L1 is complicated and diverse. This, in turn, means that in tumor-targeted therapy, the inhibition of either of these factors (PD-L1 or PD-1) alone is likely to fail in the blocking of the full PD-1/PD-L1 pathway and, hence, although it may act well in patients at lower and not-generalized stages, it is unlikely to universally help patients at all stages of the disease.

Chronic T cell activation, which may occur in persistent infections or cancer following repeated exposures to antigens, is also associated with an increased expression of inhibitory receptors such as PD-1 and CTLA-4, which leads to T cell dysfunction compromising productive T cell responses (59, 60). Yassin et al. found in experimental mice that chronic inflammation resulting in colitis-associated cancer development leads to a massive PD-1 upregulation in intraepithelial lymphocytes. This upregulation affects certain CD4 and CD8 (CD8αβ+, CD8αα+; TCRγδ+, TCRαβ+) subsets and may contribute to the reduced protective capacity of early innate (TCRγδ+) and subsequent adaptive (TCRαβ+) T cell systems (61). In addition, alterations of fecal and mucosal microbiota with reduction of bacterial species diversity have been reported in CRC patients at different cancer stages (62, 63). It follows that the immune processes themselves may be affected not only by tumor cells but also by other events taking place in adjacent areas of the tumor and which may also (negatively or positively) affect the prognosis and course of CRC. Therefore, we should not focus solely on examining the tumor, but events taking place in its vicinity need to be studied as well.

### Other Immune Processes in Colorectal Cancer Tumor Microenvironment

In addition to PD-1/PD-L1 signaling inhibition, immunotherapy relying on the combination of multiple checkpoint targets is another viable possibility for (not only) CRC treatment. Fiegle et al. showed that the dual CTLA-4 and PD-L1 blockade exerts synergistic inhibitory effects on the growth and metastasis of CRC in orthotopic mice by increasing the number of CD8^+^ and CD4^+^ T cells. This increase is associated with a Th1 response mediated by CTLA-4 inhibition and by inducing a higher number of M1 macrophages, which can mostly be ascribed to PD-L1 blockade (12). Kuwahara et al. showed that high CD4^+^ T-cell density was associated with a positive CRC outcome and constituted an independent prognostic factor in a multivariate model of CRC prognosis; its prognostic value even exceeded those of tumor invasion depth or positive lymph node metastasis (64). Previous studies have also reported that in the absence of modulation by CD4^+^ cells, specific CD8^+^ cells can become lethargic and cannot transform into long-lived functional effector cells (65, 66). However, several subsets of CD4^+^ TILs differing in their anti/protumor action were identified. One such subset, Th1 cells, activates CD8^+^ T cells, thus enhancing antitumor immunity (67). On the other hand, another subset, T helper 2 (Th2) cells, seem to suppress antitumor immunity *via* the production of immunosuppressive cytokines such as interleukin (IL)-10. A third subset, T helper 17 (Th17) cells, is reported to have both positive (facilitating antitumor immune response) and negative (increasing tumor growth by inducing tumor neoangiogenesis) effects (11). Thus, CD4^+^ T cells have a central role in managing and regulating the immune system against tumor cells.

During T cell activation, PD-1 is expressed on the surface of T cells as well as B cells and NK cells (68). Predominantly, however, it affects CD8^+^ T cells as the first line of defense against tumor cells and contributes to controlling T cell exhaustion (69). These cells, such as TILs, form the first line of the adaptive immune responses to a tumor (70). After arriving in the TME, CD8^+^ T cells should encounter both intrinsic (CD28, CTLA-4, PD-1/PD-L1, immunoglobulin-like transcript receptors), and extrinsic (regulatory T cells and myeloid cells) checkpoint regulators (71). High CD8^+^ T cell density is, therefore, another good prognostic marker in CRC, indicating an “immunologically hot” tumor microenvironment (20, 72). Reissfelder et al. highlighted the importance of TNF-α as a tissue marker of intratumoral cytotoxic T cell activity, reporting a strong correlation between the up-regulation of TNF-α expression in TILs and the total intratumoral TNF-α (73).

Also, intratumoral heterogeneity needs to be considered, in particular the differences between the tumor center and tumor margin. Kwak et al. demonstrated that the higher total number of CD3^+^ and CD8^+^ T cells in the immune infiltrate are predictive of survival. This is true for both the invasive margin and tumor center; the presence of these T cells in both these parts of the tumor further improves the prognosis (74). Miller et al. also observed an association of higher densities of PD-L1+ dendritic cells in the tumor margin with good survival (75). Dahlin et al. say that the separate measurements of T cell infiltration in the tumor margin, center, and intraepithelial compartment were closely correlated and were all positively associated with cancer-related survival (76). It follows that examining the tumor as a whole and not taking its heterogeneity into account might fail to provide us with satisfactory prognoses and results.

Some studies found that a high total lymphocyte score predicted cancer-related survival more accurately than any of the individual lymphocyte scores analyzed separately (16, 77). Ling et al. reported significantly better prognosis of patients with high levels of intraepithelial CD8^+^ T cells. Similarly, improved prognosis was reported in patients with low levels of intraepithelial CD8^+^ together with high concentration of Foxp3+ cells at the tumor invasive margin and in the centre (37). This observation was explained by the organ specific contribution of Foxp3 T cells to blocking tumor promoting inflammation stimulated by gastrointestinal bacteria, or eventually by unique cytokine profile (IL-2 and IFN-γ) or non-regulatory conventional function of at least some CRC-infiltrating Foxp3 T cells. Huang et al. also reported, compared with other subgroups, better survival outcomes in the subgroup of patients with the combination of high CD8^+^ TIL infiltration and high PD-L1 expression in tumor cells (78). Authors proposed that IFN-γ secreted by infiltrating cytotoxic CD8^+^ T cells is required for PD-L1 induction, and overexpression of PD-L1 within the tumor microenvironment acts as a negative feedback compensating for CD8^+^ CTLs and IFN-γ.

There are, of course, also other immune cells with specialized functions. NK cells are involved in the lysis of tumor cells that lose MHC class I expression (79). TME infiltration by NK alongside the CD8^+^ T cells has been also associated with improved prognosis in CRC patients (80).

B cells can modulate immune responses through various mechanisms, including inhibition of T cell responses (81). Shimabakuro-Vornhagen et al. showed that compared with the circulation of healthy individuals, the levels of highly activated and memory B cells present in the circulation of CRC patients (as well as in their TME) were elevated (82). Toor et al. showed that the total B cells levels were similar between tumor and normal tissue but in the TME, B cells expressed significantly higher levels of co-stimulatory immune checkpoints (83). This co-expression of multiple immune checkpoints can have important implications on tumor resistance to immunotherapy due to the overlap in their pathways, which may promote tumor progression (84, 85) and may be one of the reasons for the failure of immunotherapy even in patients in whom success could be otherwise.

### The Role of Innate Immune Cells

The innate immune components of the microenvironment represent the first line of defense, providing rapid response to foreign factors as well as anti-tumor activities (86). Macrophages, a component of the mononuclear phagocytic system (MPS), play a crucial role in maintaining the tissue homeostasis through regulation of the innate immune as well as inflammatory responses. Through specific differentiation, macrophages can evolve into two distinct differentiation states: classically activated M1 (proinflammatory) macrophages (87), which could promote the Th1 response, absorb and kill tumor cells (88), or alternatively, evolve into activated M2 (anti-inflammatory) macrophages, which secret anti-inflammatory cytokines such as interleukin-10 (IL-10), and promote angiogenesis, tissue remodeling, injury repair, and tumor initiation and progression (89). Upon stimulation, macrophages recruit monocytes from the blood to the tumor site and contribute to changing toward tumor-associated macrophages (TAMs) (90). In the early stage of cancer, TAMs are represented mainly by the proinflammatory M1 macrophages, and they produce reactive oxygen and nitrogen species (91); at this stage, they have antitumor effect. Later, however, tumor cells use TAMs to support CRC and (through multiple signaling pathways) convert them into anti-inflammatory and cancer-promoting M2 phenotypes with tumorigenic activity (92). The role of TAMs in CRC is, therefore, highly complicated and their simple presence or absence is not a clear indicator of the patient’s prognosis and overall survival. It, however, appears that their presence in the early stages of cancer is rather a positive prognostic marker while in the later stages, it affects the prognosis negatively.

Dendritic cells (DCs) are professional antigen-presenting cells and their interrupted development allows tumor cells to evade immune recognition. To avoid immune surveillance, cancer cells may suppress DCs through multiple mechanisms, including the secretion of immunosuppressive TGF-β or IL-10 (93). It was reported that these DCs secrete increased levels of immunosuppressive IL-10 and decreased levels of immunostimulatory IL-12 and TNF-α (94) or the chemokine (C-X-C motif) ligand 1(CXCL1), which enhances tumor cell migration (95). Several studies reported that the number and functions of blood DC subsets were reduced in CRC patients, demonstrating that the range of these effects correlated with the disease stage and prognosis (94, 96). Another point of view is offered by the work of Miller et al., which demonstrated that PD-L1+ tumor-associated dendritic cells correlate with improved survival in CRC patients, which may reflect an active T cells-driven anti-tumor immune response (75). These observations are, however, not mechanistically reliably explained yet and the situation is similar to TAMs.

Mast cells (MCs) are multifunctional cells whose role is to participate in allergic and infectious events. In cancers, their presence has been described mainly at the tumor margins and at peri-vascular regions. It was associated with increased blood vessel density in the TME (97) and with poor prognosis (98). According to Zhao et al., a lower frequency of circulating MCs and their progenitors was found in patients with CRC than in healthy patients; this was especially apparent in patients with advanced disease. It turned out that this may reflect aggressive CRC course (99).

Myeloid-derived suppressor cells (MDSC) represent a heterogeneous population of granulocytes and monocytes that rapidly expand during infection, inflammation, and cancer (100). They have been reported to suppress T cell response through reactive oxygen species (ROS) inducing antigen-specific tolerance as well as through elevating inducible nitric oxide synthase (iNOS), arginase-I (ARG1) and other suppressive cytokines (101, 102). Their recruitment depends on various CRC-derived mediators including chemokines, with chemokine (C-C motif) ligand 2 (CCL2) being particularly relevant. MDSC recruited by the CCL2-CCR2 (C-C chemokine receptor type 2) signaling pathway in mice models were shown to support CRC growth (103). Their relevance for predicting patient prognosis, however, remains unknown. Innate immunity cells contribute to the protection from tumor cells proliferation particularly at early stage of tumor development. At the clinically manifested stage their protective effect is substantially limited.

It follows that most of the immune cells may actually have a dual activity—anti- and pro-tumor, depending on the signals received from the TME. These signals may support anti-tumorigenic functions or modulate immune cells into a protumorigenic phenotype. This knowledge reflects the potential and importance of the role of TME and the potential of treatment approaches targeting TME for the treatment of CRC. As an example, we can mention therapies blocking CTLA-4, PD-1/PD-L1 pathways, those limiting monocyte infiltration, reprogramming polarization of TAMs, blocking secretion of immunosuppressive TGF-β or IL-10 by cancer cells, or inhibiting the activity of MDSCs.

### Tumor Microenvironment and Immune Response to Colorectal Cancer

Various mechanisms have been proposed to explain how the TME contributes to tumor progression, tumor invasion, and metastasis. For instance, the following mechanisms were proposed: 1) impacting the proliferation and survival of cancer cells; 2) increasing their stem-like properties and favoring epithelial-to-mesenchymal transition (EMT); (104–106), 3) rewiring the tumor metabolism (107) and/or 4) stimulating metastatic dissemination (108). EMT was shown to be a pivotal driver of fibrosis development in embryonic tissues, wound healing, tumorigenesis, and metastasis (109). It is a dynamic process involving not only pure epithelial or mesenchymal cells but also “hybrid” cells (110). Previous studies have suggested EMT activation in a number of malignant tumor cells, resulting in the loss of typical characteristics of epithelial cells (cell-cell junctions and apical basal polarity), and acquiring properties of mesenchymal cells, which in turn promotes migration and invasion-metastasis cascade (109, 111). Cancer cells with mesenchymal phenotypes are also less susceptible to cytotoxic lymphocyte-mediated lysis and NK cell attacks (112). EMT is mediated by immune cells in the TME secreting cytokines, inflammatory factors, and chemokines. Also, cancer cells can crosstalk with immune cells to induce cell plasticity and release immunosuppressive substances, thus creating an immunosuppressive microenvironment that promotes invasion and metastasis (113, 114).

Cancer-associated fibroblasts (CAFs), non-neoplastic cells present in the tumor, are a major contributor to EMT. CAFs produce desmoplastic reaction (DR), commonly observed in invasive CRC. Desmoplasia is the rearrangement of organized, anisotropic extracellular matrix fibers in a pathological microenvironment associated with metastasis or poor patient survival (115). Immature desmoplasia is characteristic of tumors with keloid-like collagen or myxoid stroma, which have been associated with poor survival in CRC patients (116). Periostin (POSTN), as a product of CAFs, could be another key molecule contributing to the malignant potential of CRC by modifying the desmoplastic environment (117). CAFs in tumor tissue also antagonize the T-cell antitumor activity and negatively contribute to patients’ prognosis (118). Monitoring of desmoplastic reactions, therefore, appears to be a potentially promising prognostic parameter.

## Immunoscoring

Evaluation of the amount and type of TIL seems to be a suitable complement to the standard TNM classification, especially when deciding on adjuvant therapy. The immunoscore (IS) methodology quantifies and detects various types of immune cells in tumor tissue, and, besides, determines the density of their infiltration and localization in the center and invasive margin. It offers ratings from 0 (low immune system cell infiltration in both areas) to 4 (high immune system cell infiltration in both areas). There are two advantages to this examination: 1) IS appears to be a strong prognostic factor for disease-free survival and overall survival and 2) it has biological significance (adaptive and also an innate immune response to the presence of tumor cells). It can, therefore, be also used as a tool for the management of therapy, including immunotherapy (22, 119–121). In addition, adaptive cell therapy using TILs from patients, donors, or differentiated from stem cells, is a highly promising immunotherapeutic strategy in CRC patients. These immune cells are activated and expanded *in vitro* (and, if need be, possibly subjected to gene modifications) before finally being administered to the patient (122). *Beak* and *Kim* obtained TILs from patients with CRC and evaluated their potential as an immunotherapeutic modality. They demonstrated that the *ex vivo* expanded TILs contained mostly the effector memory T cell subset and elicited anti-tumor response (123). Koelzer et al. have demonstrated CD8^+^ and CD45RO + memory T cell infiltration in preoperative biopsies to be reliable independent prognostic markers associated with abundant biological behavior after resection (independently of the TNM stage and postoperative therapy) (Table 1). The high level of this infiltration predicts a locally less advanced tumor along with an absence of nodal metastases and lymphovascular invasion (124).

**TABLE 1:** Landmark studies indicating the value of different cell populations in predicting CRC prognosis.

Cell population	Author	Cancer location	TNM stage	Sample size	Main results
CAF	Akishima-Fukasawa et al. (131)		I–III	110	PGP9.5 expression is an independent prognostic factor for overall and recurrence-free survival.
CAF	Glentis et al. (132)	CRC and adenoma	Not defined	40	CAFs actively assist cancer cells to breach the basement membrane.
CAF	Ren et al. (133)	CRC	Not defined	not defined	CAFs promote the stemness and chemoresistance of CRC by transferring exosomal H19.
CAF	Zhang et al. (134)	CRC	Not defined	not defined	Colorectal-cancer-CAFs-derived HGF induced up-regulation of CD44 which mediated adhesion of CRC cells to endothelial cells, and subsequently resulted in enhancement of metastasis of CRC.
CAF	Miyazaki et al. (135)	CRC and breast cancer	Not defined	not defined	The direct interaction with CAFs, as well as environmental cytokines, contributes to the collective invasion of cancers.
CAF	Unterleuthner et al. (136)	CRC	Not defined	not defined	WNT2 has a pivotal role in sustaining an activated CAF phenotype, which is associated with the maintenance of a pro-angiogenic secretome and contributes to elevated tumor angiogenesis in CRC.
DC	Bauer et al. (137)	CRC (MSI-H and MSS)	Not defined	69	Impaired DC maturation may contribute to local immune evasion in CRC.
DC	Gulubova et al. (138)	CRC	I - IV	145	The infiltration of colon cancer with DCs is related to tumor progression and patient prognosis, suggesting a central role of DCs in controlling local tumor immunity.
DC	Hu et al. (139)	CRC	not defined	19	Treatment with anti-PD-L1 may promote the maturation of Dcs and enhance the functionality of DC1 subtype.
DC	Miller et al. (75)	CRC	III	221	PD-L1-expressing DC in the tumor microenvironment are associated with improved survival in stage III colon cancer and likely reflect an immunologically “hot” tumor microenvironment.
B cells	Berntsson et al. (140)	CRC	I–IV	557	Dense infiltration of CD20^+^ B-cells is an independent predictor of a favourable clinical outcome.
B cells	Edin et al. (141)	CRC	I–IV	316	There is a positive prognostic role of tumour-infiltrating CD20^+^ B lymphocytes in CRC patients.
B cells	Mullins et al. (142)	CRC and rectal cancer	III–IV	25	Tumor-infiltrating B cells can contribute to tumor control in a dula role of sole antigen-presentation and additionally anti-tumoral Ig-production.
B cells	Toor et al. (83)	CRC	I–IV	50	Decreased levels of B cells and selective IC-expressing CD8^+^ TILs are associated with tumor progression. MSI-H tumors could show favorable prognosis/improved response to cancer immunotherapy.
T cells	Li et al. (51)	CRC	I–IV	356	Higher expressions of PD-1 and PD-L1 correlates with a better prognosis in CRC patients.
T cells	Berntsson et al. (143)	CRC	I–IV	557	A high density of cytotoxic T cells is an independent prognostic factor in right-sided tumours and regulatory T cells predict longer survival only in patients with rectal tumours.
T cells	Digiacomo et al. (144)	BRAF-mCRC	IV	59	A simultaneous evaluation of MSI, CD8 T-cell content, and neuroendocrine markers could allow for the identification of subsets of BRAF-mCRC with a different prognosis and potential eligibility for specific treatments.
T cells	Glaire et al. (145)	CRC	II–III	1804	The prognostic value of intratumoral CD8^+^ cell infiltration in stage II/III CRC varies across tumour and nodal risk strata.
T cells	Fiegle et al. (12)	Mouse model of CRC		25	Dual CTLA- and PD-L1 blockade exert synergistic inhibitory effects on growth and metastasis of the orthotopic CT26 colon tumors by increasing CD8^+^ and CD4^+^ T cells.
T cells	Kuwahara et al. (64)	CRC	I–IV	342	Intratumoral CD4^+^ T-cell density and combined CD4^+^ and FOXP3+ T-cell densities were stronger prognostic factors than other clinicopathological features.
T cells	Craig et al. (146)	CRC	II–IV	1724	Immune cold patients by assessment of CD3, CD4 and CD8 IHC are linked with difficult-to-treat, poor prognosis hypoxic biology, which may be potentially amenable to targeted therapy or monitoring for disease progression.
T cells	Noh et al. (48)	CRC	I–IV	489	Tumours with PD-L1-positive tumour cells and high-CD8 TIL is associated with the best prognosis, and show stronger CD8/PD-L1/Pd-1 signalling interaction compared to the other types.
T cells	Hartman et al. (147)	CRC	I–IV	259	The prognostic value of MMR protein deficiency is most likely attributed to increased tumor-associated CD8-positive T cells and that automated quantitative CD8 T-cell analysis is a better biomarker of patient prognosis.
T cells	Fuchs et al. (148)	CRC	I–IV	1034	ITWG systém for assessing TILs is a powerful predictor of all-cause survival in CRC independent of many prognostic factors and superior to the assessment of intraepithelial lymphocytes using a traditional system.
T cells	Lalos et al. (149)	CRC	I–IV	613	The combination of high CD8^+^ T-cell density and expression of SDF-1 represents an independent, favorable, prognostic condition in CRC, mostly in patients with stage III disease.
T cells	Al-Badran et al. (150)	CRC	I–III	773	Individual and combined high expression of TIM-3, LAG-3, and PD-1 on stromal immune cells are associated with better colorectal cancer prognosis.

The predictive potential of IS has been reported in several studies. For example, Morris et al. reported longer overall survival in patients with TILs who received adjuvant chemotherapy based on 5-fluorouracil (125). Similarly, the high density of CD3^+^, CD8^+^ and granzyme B+ lymphocytes in the invasive margin of hepatic CRC metastases was predictive of prolonged disease-free survival in patients treated with conventional chemotherapy or chemotherapy in combination with cetuximab or bevacizumab (126). However, more studies and more patients will be needed for validating and improving the IS sufficiently to be used in routine clinical practice.

### Consensus Molecular Subtypes

The analyses of the effects of the immune infiltrate in specific subgroups of patients and their clinical outcome led to the development of a new classification system based on the consensus between different classification systems proposed by various research groups. Thus, four “consensus molecular subtypes” (CMSs) of CRC were defined (8): CMS1 is characterized by MSI, mutations in CIMP and BRAF pathways, a diffuse immune infiltrate, and strong activation of immune evasion pathways. CMS2 tumors show high chromosomal instability and activation of *Wnt* and *MYC* pathways. CMS3 tumors exhibit frequent *KRAS* mutations and disrupted metabolic pathways, and CMS4 tumors are characterized by high expression of mesenchymal genes, stromal infiltration, angiogenesis, and TGF-β activation. CMS1 and CMS4 are tumors characterized by strong immune infiltration while, on the contrary, CMS2 and CMS3 are tumors without immune activation. Their prognoses also differ, with CMS4 tumors displaying worse overall and relapse-free survivals (8). This classification provides an interesting opportunity to explore the heterogeneity of CRC. Understanding CMS is a crucial step towards personalized medicine as it could optimize the management of individual patients. For example, it will allow defining the best first-line chemotherapy regimen (poor efficiency of oxaliplatin in CMS1 and CMS4) or application of immunotherapy for metastatic CRC in CMS4 patients (127).

The biological and prognostic associations of CMS in the context of metastatic tumor heterogeneity is slightly more complicated. Eide et al. performed exploratory analyses of a dataset of 317 primary tumor samples and 295 liver metastasis samples. Almost 90% of metastases belonged to CMS2 or CMS4 subtypes, which is in line with the aforementioned poorer prognosis of these two subtypes (128). These results were further corroborated by Fontana et al. and Kamal et al. (129, 130). The difference is caused by a combination of biological intrinsic and extrinsic factors, including the reaction to treatment; for example, metastatic tumors after chemotherapy have been shown to tend towards CMS4 classification, even if the primary tumor was another subtype with originally better prognosis (such as CMS3) (130).

However, it must be considered that even within CMS groups, there is high inter- and intratumor heterogeneity, which limits universal application of the CMS classification in the clinical practice as a predictive prognostic marker. In view of this, further subclassification based, for example, on detailed gene expression profiling methods like single-cell RNA sequencing and analyses might refine this classification system and improve its clinical usability.

## Conclusion

This review shows us that despite the large amount of available information and relatively detailed knowledge of the immune mechanisms that take place inside the tumors and in their vicinity, many factors and variables remain unclear, which ultimately determine success of proposed therapy. The immune contexture of each CRC is highly relevant for the design and allocation of therapeutic approaches that depend on anti-tumoral immune responses and include conventional therapeutic options (chemo- and radiotherapy) as well as immunotherapy. PD-L1+ positive tumor cells and CD8^+^ TIL are, therefore, key prognostic biomarkers for locally advanced CRC patients treated with neoadjuvant chemoradiotherapy. Upregulation of immune response with immunotherapy has been successful in a subset of patients with dMMR/MSI-H CRC patients. Choosing the right combination of drugs and the right therapeutic strategy for each individual patient including those with metastasized CRC is the key to successful therapy. These combinations and individualization will be crucial aspects of a future clinical trial also at our department. Future directions in the study of immunotherapy in CRC will include identifying improved biomarkers in patients with pMMR/MSS, and identifying novel immunotherapies with improved efficacy, such as specific treatments targeting innate immune cells supporting tumor growth.

## Data Availability

The original contributions presented in the study are included in the article/supplementary material, further inquiries can be directed to the corresponding author.
